# Ability of presepsin concentrations to predict mortality in adult patients with sepsis

**DOI:** 10.1017/cts.2023.538

**Published:** 2023-05-03

**Authors:** Ajete Aliu-Bejta, Mentor Kurshumliu, Sadie Namani, Shemsedin Dreshaj, Bruno Baršić

**Affiliations:** 1 University Clinic of Infectious Diseases, Alexander Fleming, Pristina, 10000, Kosovo; 2 University of Pristina “Hasan Prishtina”, Faculty of Medicine, Lagja e spitalit, p.n, Pristina, 10000, Kosovo; 3 “PROLAB” Biochemical Laboratory, Mark Dizdari, Pristina, 10000, Kosovo; 4 University of Zagreb, School of Medicine, Šalata 4, Zagreb, 10000, Croatia; 5 University Hospital for Infectious Diseases “Dr. Fran Mihaljević,” Zagreb, 10000, Croatia

**Keywords:** Presepsin, PCT, CRP, SOFA, APACHE II

## Abstract

**Background:**

Early diagnosis of sepsis is essential for a favorable disease outcome. The aim of this study was to evaluate the association of initial and subsequent presepsin concentrations with sepsis outcomes.

**Methods:**

One hundred sepsis patients were enrolled in the study from two different university centers. Four times during study, concentrations of presepsin, procalcitonin (PCT), and C-reactive protein (CRP) were measured, and Sequential Organ Failure Assessment (SOFA) score and Acute Physiology and Chronic Health Evaluation (APACHE II) score were calculated. Patients were grouped into survivors and nonsurvivors. A sandwich ELISA kit was used to measure presepsin concentrations. To test the changes in biomarkers concentrations and SOFA score and APACHE II score during the disease course and to estimate the differences between outcome groups, generalized linear mixed effects model was used. Receiver operating characteristic curve analysis was performed to determine the prognostic value of presepsin concentrations.

**Results:**

Initial values of presepsin, SOFA score, and APACHE II score were significantly higher in nonsurvivors compared to survivors. Concentrations of PCT and CRP did not differ significantly between outcome groups. ROC curve analyses show a greater predictive ability of initial presepsin concentrations for predicting mortality compared to subsequent measurements of presepsin concentrations.

**Conclusions:**

Presepsin has a good ability to predict mortality. Initial presepsin concentrations better reflects poor disease outcome compared to presepsin concentrations 24 and 72 hours after admission.

## Introduction

Sepsis is a condition that complicates severe infections and is a leading cause of death in critically ill patients [1–3]. Rudd *et al*. [4], in an epidemiological study, reported sepsis-related deaths worldwide to be around 20% of all recorded deaths in 2017. Incidence of sepsis has increased over decades [5,6]. Better recognition of sepsis, advancement of medicine, as well as increased use of immunosuppressive therapy, and growing number of people living with chronic comorbidities and transplants, may influence the increase of incidence of sepsis.

Early diagnosis of sepsis is essential for a favorable disease outcome. Finding a biomarker that is specifically increased in septic patients at the early stage of disease which has a good prognostic capacity is crucial for early identification and prompt treatment of critically ill patients. Various biomarkers have been introduced to enable a rapid evaluation of critically ill patients [7]. Among them, soluble CD14 subtype (sCD14-ST), also known as presepsin, seems to be a promising biomarker for early diagnosis of sepsis. Presepsin, first identified in 2005, was reported as a marker specifically increased in patients with sepsis [8].The prognostic ability of presepsin was also evaluated. Presepsin was reported to be useful for mortality prediction [9–17]. A correlation between initial presepsin values and in-hospital mortality in patients with sepsis was previously described [9,10,14–16,18,19]. In a multicenter randomized Albumin Italian Outcome Sepsis trial (ALBIOS) [12], it was reported that presepsin levels remained high over 7 days in nonsurvivors and decreased over time in survivors. Higher initial presepsin levels were associated with mortality. In 2014, Masson *et al*. compared prognostic accuracy of presepsin and procalcitonin (PCT) in mortality prediction; they found presepsin to be a marker of mortality with better prognostic performance than PCT [13]. The aim of this study was to evaluate at what timepoints presepsin best predicts poor disease outcome.

## Materials and Methods

We conducted a prospective observational study in two university clinical centers: University Clinical Center of Kosovo, Clinic of Infectious Diseases in Pristina, Kosovo, and University Clinical Center of Zagreb, Hospital for Infectious Diseases in Zagreb, Croatia. Patients’ enrolment was done in two study periods, after obtaining informed consent from patients or their supervisor. Patients enrolled in the study were treated in the intensive care unit of both hospitals, and a small number of patients were treated in the Department of Neuro-infections and Blood-stream Infections at the Clinic of Infectious Diseases, in Pristina. Between February 2015 and May 2016 in the study were enrolled patients admitted at the Clinic of Infectious Diseases in Pristina, whereas during 2018, in the study were enrolled patients admitted at the Hospital for Infectious Diseases in Zagreb, Croatia.

The study was compliant with the 2008 Helsinki Declaration. Prior to the start of the study, the ethical approval was obtained from the Ethics Committee of both University Clinical Centers, in Pristina and in Zagreb.

### Patients’ Inclusion and Exclusion Criteria

Clinical criteria determined by Sepsis-3 Consensus Conference were used for patient’s inclusion in the study [20]. All patients suspected for sepsis aged 18 years and over who fulfilled at least two of the qSOFA (quick Sequential Organ Assessment screening tool) criteria: altered mentation, systolic blood pressure < 100 mmHg, and respiratory rate > 22 /min were enrolled in the study. Patients younger than 18 years were excluded from the study. After initial inclusion in the study, when a diagnosis different than sepsis was evident, patients were later excluded from the study.

### Patents’ Grouping

Patients were grouped according to disease outcome into survivors and nonsurvivors. Patients were followed until ICU or hospital discharge. Death was considered as an unfavorable outcome.

### Data and Sample Collection

Clinical and laboratory parameters needed for calculating APACHE II score, as well as blood samples for measuring C-reactive (CRP), PCT, and presepsin, were collected four times during the study: on admission (T0), after 24 hrs (T1), and after 72 hrs (T2), and on Day 7 (T3). For PCT and presepsin measurements, blood samples were collected and frozen until the end of study, then measured. Blood samples for measuring presepsin and PCT concentrations were collected on test tubes with anticoagulants (ethylenediamine tetraacetic acid-EDTA or sodium citrate) and centrifuged for 15 minutes at 1000 × g, 30 minutes within collection, than serum samples were stored at –40°C until the end of study. PCT concentrations were measured in both centers: Institute of Biochemistry, Pristina, University Clinical Center of Kosovo, and University Hospital for Infectious Diseases in Zagreb, Croatia. Quantitative analysis of PCT was performed using an automated electrochemiluminescence immunoanalyzer (ELECSYS* BRAHMS* PCT; Roche Diagnostics, Mannheim, Germany). Concentrations of presepsin were measured in “PROLAB” biochemical laboratory in Pristina, using a sandwich enzyme-linked immune-sorbent assay-Human Presepsin ELISA Kit. ELISA kits for presepsin measurement were imported from the manufacturer Nordic Biosite based in Sweden, after obtaining permission for import from Agency for Medicinal Products of Kosovo. Kits were used for research purposes only.

### Statistical Analysis

Categorical variables were reported as frequency and percentage. Continuous variables were reported as means ± one standard deviation (±SD). Simple comparisons were made for categorical variables using the chi-square test or Fisher’s exact test as appropriate, and the Wilcoxon rank-sum test for continuous variables. Generalized linear mixed effects model was used to test the changes in biomarkers concentrations during the disease and to estimate the difference between two outcome groups (survivors and nonsurvivors) after adjustments for baseline biomarkers values. Longitudinal analysis using generalized linear mixed effects modeling was performed to test the association of initial values of SOFA score and APACHE II score with poor outcome, after adjustment for initial values and day of illness. Adjustment was done because baseline score values have a strong impact on subsequent values. To estimate and to compare the associations of initial and subsequent values of presepsin with poor disease outcome, we constructed the ROC curves and calculated the AUCs. Significance was set at an alpha level of 0.05, for all statistical tests. All analyses were performed using SAS software version 9.3 (SAS Institute, Cary, North Carolina, USA).

## Results

### Demographics and Clinical Characteristics of Enrolled Patients

Out of 116 patients initially included in the study, 16 were later excluded due to the evidence of a diagnosis different than sepsis: Hantan hemorrhagic fever, carcinomatous meningitis, leptospirosis, etc. Finally, one hundred sepsis patients (48 males, 52 females) were enrolled in the study. There were 68 survivors, whereas 32 patients died during the course of the disease. There were no significant differences in mortality related to age and gender. Mean age of patients who died during the disease course was 66.8 years (SD ± 14.0), whereas mean age of surviving patients was 59.7 years (SD ± 17.2) (*p* = 0.066). We found higher mortality in patients with pulmonary site of infection (*p* < 0.004) and those with chronic obstructive pulmonary disease (*p* = 0.028) (Table 1).

**Table 1.** Baseline clinical and laboratory characteristics of enrolled patients

Characteristics	Non-survivors	Survivors	*P* value
Age (mean±SD)	66.8 (±14.0)	59.7 (±17.2)	0.066
Gender			0.188
Female (no, %)	13 (40.6%)	39 (57.4%)	
Male (no, %)	19 (59.4%)	29 (42.6%)	
**Site of infection (no, %)**			
Respiratory tract	17 (53.1%)	23 (33.8%)	0.004
Genitourinary tract	1 (3.1%)	26 (38.2%)	0.002
Intra-abdominal	3 (9.4%)	5 (7.4%)	0.728
Skin and soft tissues	4 (12.5%)	4 (5.9%)	0.255
Other	7 (21.9%)	10 (14.7%)	0.019
**Chronic comorbidities (no, %)**			
Diabetes	15 (46.9%)	29 (42.6%)	0.691
COPD	6 (18.8%)	3 (4.4%)	0.028
Regular hemodialysis	2 (6.3%)	5 (7.4%)	0.840
Recent surgery	3 (9.4%)	4 (5.9%)	0.685
No evident risk factor	10 (31.3%)	25 (36.8%)	0.589
**Scoring systems (mean±SD)**			
SOFA score	10.0 ± 3.3	5.6 ± 2.8	< 0.001
APACHE II score	30.4 ± 9.7	20.3 ± 7.9	< 0.001
**Biomarkers (mean±SD)**			
Presepsin (ng/ml)	121.1 ± 52.6	93.2 ± 65.5	0.028
Procalcitonin (ng/ml)	22.0 ± 32.1	25.5 ± 33.4	0.742
C-reactive protein (mg/ml)	228.4 ± 149.9	198.1 ± 95.2	0.537

APACHE II, acute physiology and chronic health evaluation; COPD, chronic obstructive pulmonary disease; SD, standard deviation; SOFA, sequential organ failure assessment.

We previously published the results of our study regarding the accuracy of presepsin in identifying sepsis patients and its correlation with SOFA score, a score designed to identify sepsis patients [21]. Here, we will present the association of presepsin concentrations with mortality.

### Scoring systems and biomarkers values on admission

Mean values of measured biomarkers (presepsin, PCT, and C-reactive protein), SOFA score, and APACHE II score in two outcome groups on admission are presented in Table 1.

Mean values of presepsin, SOFA score, and APACHE II score on admission were significantly higher in nonsurvivors compared to survivors, 121.1 ± 52.6 ng/ml vs 93.2 ± 65.5 ng/ml (*p* = 0.028); 10 ± 3.3 vs 5.6 ± 2.8 (*p* < 0.001); 30.4 ± 9.7 vs 20.3 ± 7.9 (*p* < 0.001), respectively. The same was not found for PCT and CRP. Initial values of PCT (22.0 ± 32.1 vs 25.5 ± 33.4 ng/ml (*p* = 0.742)) and CRP (228.4 ± 149.9 vs 198.1 ± 95.2 mg/ml (*p* = 0.537)) did not differ significantly between nonsurvivors and survivors.

### Kinetics of Presepsin, PCT, CRP, SOFA Score, and APACHE II Score During Study

**Figure 1.**
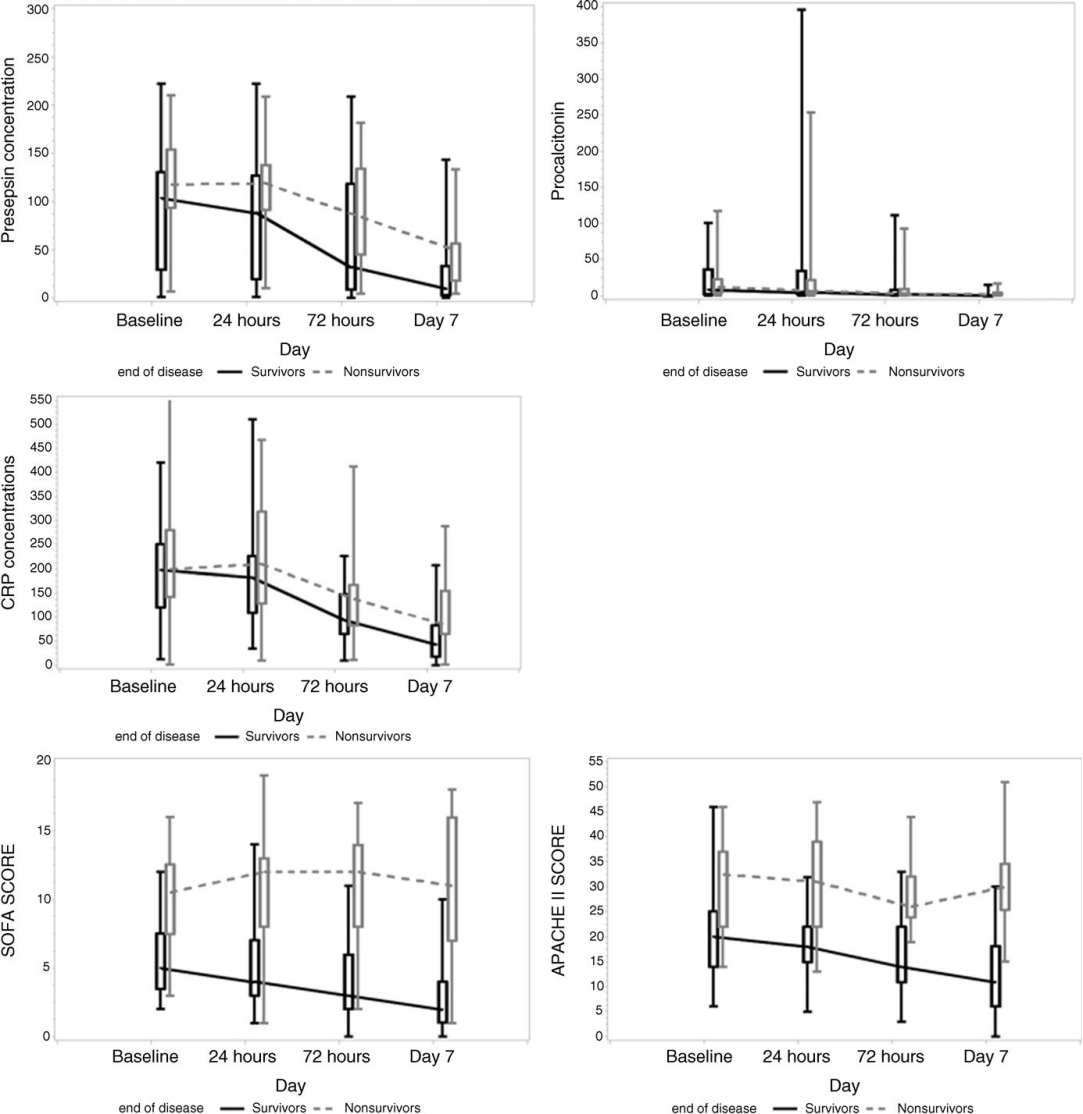
Kinetics of sepsis biomarkers, SOFA score, and APACHE II score throughout the study. Concentrations of measured sepsis biomarkers (presepsin, PCT and CRP, and scoring systems (SOFA and APACHE II) are presented at four time points (T0, T1, T2, T3). APACHE II, acute physiology and chronic health evaluation II; CRP, C-reactive protein; PCT, procalcitonin; SOFA, sequential organ failure assessment; T0, day of admission; T1, after 24 hrs; T2, after 72 hrs; T3, on day 7.

Dynamics of presepsin, PCT, CRP, SOFA score, and APACHE II score are presented in Table 2 and Fig. 1. Presepsin concentrations were significantly higher in nonsurvivors compared to survivors at all time periods (T0–T3), suggesting its possible prognostic ability. Higher initial presepsin concentration and its slower decrease were associated with increased mortality. Initial and subsequent values of PCT and CRP did not differ significantly between nonsurvivors and survivors. SOFA score and APACHE II score were significantly higher in nonsurvivors compared to survivors on admission and at every time point. Both scores were significantly higher in nonsurvivors compared to survivors on admission, and they remained high or even increased in nonsurvivors at the following time points.

**Table 2.** Dynamics of biomarkers values, SOFA score, and APACHE II score during study

	non-survivors	survivors	P value
**T0 (on admission) (mean±SD)**			
Presepsin (ng/ml)	121.1 ± 52.6	93.2 ± 65.5	0.028
PCT (ng/ml)	23.2 ± 32.2	25.5 ± 53.6	0.742
CRP (mg/ml)	228.4 ± 149.9	198.1 ± 95.2	0.537
SOFA score	10.0 ± 3.3	5.6 ± 2.8	< 0.001
APACHE II score	30.4 ± 9.7	20.3 ± 7.9	< 0.001
**T1 (after 24 hrs.) (mean±SD)**			
Presepsin (ng/ml)	116.2 ± 51.4	85.0 ± 64.7	0.039
PCT (ng/ml)	28.4 ± 54.1	24.9 ± 53.6	0.773
CRP (mg/ml)	216.4 ± 121.8	176.1 ± 82.2	0.117
SOFA score	10.8 ± 4.2	5.0 ± 3.0	< 0.001
APACHE II score	30.6 ± 10.2	18.4 ± 6.5	< 0.001
**T2 (after 72 hrs.) (mean±SD)**			
Presepsin (ng/ml)	85.4 ± 53.6	58.0 ± 56.7	0.047
PCT (ng/ml)	13.9 ± 27.8	6.9 ± 15.7	0.306
CRP (mg/ml)	161.3 ± 122.5	108.6 ± 58.3	0.092
SOFA score	11.3 ± 4.6	4.0 ± 2.7	< 0.001
APACHE II score	28.9 ± 7.6	15.7 ± 7.1	< 0.001
**T3 (on Day 7) (mean±SD)**			
Presepsin (ng/ml)	46.6 ± 37.4	22.7 ± 31.1	0.010
PCT (ng/ml)	3.3 ± 4.7	1.3 ± 2.4	0.185
CRP (mg/ml)	111.5 ± 76.8	57.2 ± 46.8	0.034
SOFA score	11.3 ± 5.3	2.8 ± 2.6	< 0.001
APACHE II score	30.7 ± 9.8	12.0 ± 7.1	< 0.001

APACHE II, acute physiology and chronic health evaluation; CRP, C-reactive protein; PCT, procalcitonin; SD, standard deviation; SOFA, Sequential Organ Failure Assessment.

### Predictive Values of Presepsin on Poor Disease Outcome

**Figure 2.**
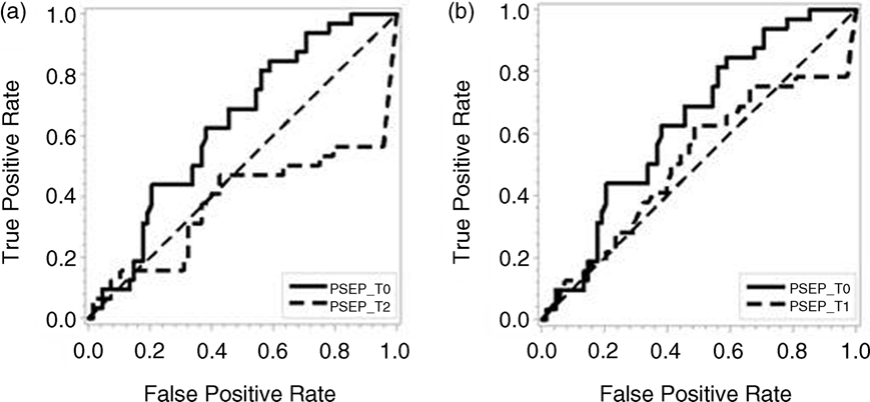
ROC curves of presepsin values on admission compared to curves after 24 (A) and 72 (B) hours regarding mortality.

We evaluated predictive values of presepsin concentrations on admission and after 24 and 72 hours. Subsequent measurements are compared to initial values on admission. The Bonferroni correction of p-values was used to counteract the problem of multiple comparisons of presepsin values on admission and after 24 and 72 hours, so values lower than 0.025 were considered significant. Results of ROC curve areas and comparison of curves at different times regarding initial values are presented in Table 3 and Fig. 2. Our results show that initial presepsin values have greater predictive value on mortality than subsequent measurement.

**Table 3.** ROC curve areas and 95% confidence intervals for presepsin values on admission, after 24 and 72 hours

Timing of presepsin sampling	ROC Area	Confidence	Limits	*p*-value*
**On admission**	0.6365	0.5254	0.7476	
**After 24 hours**	0.6406	0.5239	0.7573	0.8939
**After 72 hours**	0.6540	0.5204	0.7877	0.7044

* Subsequent measurement compared to initial presepsin value.

Predictive values of presepsin levels on mortality did not differ regarding the time of sampling.

## Discussion

Elderly people are more likely to develop sepsis, probably due to the impaired age-related immunologic defence as well as the accompanying comorbidities that *per se* increase the risk for infection. More than half of patients with sepsis are ≥ 60 years old [22–26]. Males are more likely to die from sepsis than females [23–27]. In the present study, 68% of patients were ≥ 60 years old. Higher mortality was observed among male patients with sepsis compared to female patents, 59.4% vs 40.6%, respectively. In our study, we found a small predominance of females (52%). A slight predominance of female patients with severe sepsis was also found by Angus *et al*. in a large observational cohort study on 192,980 severe sepsis patients [23]. As previously reported in other studies [11,22–24,26,28–30], we also found the respiratory tract to be the most common site of infection. Regarding chronic accompanying comorbidities, we found COPD as the only risk factor associated with death (*p* = 0.028). Degoricija *et al*. have also reported that COPD has an impact on sepsis outcome [27].

Concentrations of sepsis biomarkers (presepsin, PCT, and CRP) were evaluated on admission and during the course of the disease. We found significantly higher presepsin concentrations on admission and at every subsequent measurement in nonsurvivors compared to survivors. The same was not found for PCT and CRP. The higher was the initial presepsin concentration, and the worst was the disease outcome. Differences in initial presepsin values between survivors and nonsurvivors and its association with mortality from sepsis, was previously observed in other studies [9–13,31–33]. Our results confirmed the ability of initial presepsin concentrations in patients with sepsis to predict mortality.

In the present study, we found different trends of presepsin concentrations over the disease course between nonsurvivors and survivors. Presepsin concentrations decreased slowly and remained high until Day 7 in nonsurvivors, at least 4-fold above the upper reference range, while in patients who survived presepsin concentrations decreased more rapidly and returned within the reference range on Day 7. PCT levels decreased in a similar way in nonsurvivors and survivors. Similar findings were previously reported [11–13]. The decreasing trend of presepsin concentrations in patients who survived may be attributed to the recovery of kidney function and the appropriateness of antibiotic therapy. It was previously reported that kidneys are an important organ for presepsin clearance [34]. We can speculate that in surviving patients, presepsin concentrations decreased earlier due to the appropriateness of antibiotic therapy and the rapid recovery of kidney function, whereas nonsurviving patients had severely impaired kidney function. The decrease of absolute value of presepsin was also noted in nonsurviving patients. Production of presepsin is related to bacterial phagocytosis [35]. The decreasing trend of absolute value of presepsin observed in nonsurvivors may be due to the lack of stimulation of presepsin production. Even when bacterial infection is defeated, critically ill sepsis patients may die from the multiple organ dysfunction caused by the powerful inflammatory response.

Contradictory results have been reported by different studies regarding the usefulness of PCT for mortality prediction. In our study, values of PCT did not differ significantly between nonsurvivors and survivors on admission or at any other subsequent measurement. Although significantly higher initial PCT concentrations in nonsurvivors compared to survivors were already reported in previous studies, an association between initial PCT levels and mortality was not found [10,13,36]. Presepsin was found to have a better mortality prediction ability compared to PCT [9,13,32,33].

In our study, both scores, SOFA score and APACHE II score, as expected, were significantly higher in nonsurvivors compared to survivors, which is in line with previously published results [9,11,13,31–33,37–40].

To compare the predictive value of presepsin concentrations for mortality from sepsis, ROC curves for presepsin concentrations at different time points were constructed and compared. We found presepsin to be a good mortality predictor. Initial presepsin concentrations have a greater ability to predict mortality in comparison to subsequent levels of presepsin measured 24 and 72 hours after admission. To our knowledge, this is the first study to report the ability of presepsin to predict mortality by comparing presepsin values at different time points during study. Our study highlights the adequacy of measuring and comparing baseline presepsin values between survivors and nonsurvivors, since our results show that initial presepsin values best predicted mortality.

Bosch *et al*. [18], in a prospective study on 31 patients with intra-abdominal site infection undergoing emergency surgery, reported that initial presepsin concentrations had the highest AUC predicting mortality, compared to IL-6, PCT, endotoxin, CRP, and white blood cell count, although initial presepsin concentrations were not associated with 90-day mortality. Zhu *et al*. [41], in a meta-analysis recently published, reported an acceptable prognostic accuracy of presepsin for predicting mortality from sepsis. The same study reported PCT to be a reliable biomarker in the prognostic value of mortality. Both markers were able to distinguish nonsurvivors from survivors. Clementi *et al*. [42], in a study on 122 cardiac surgery patients, measured levels of presepsin and PCT 48 hours after cardiac surgery and compared the predictive value of both markers. They found presepsin to have a better predictive value for in-hospital, 30 days, and 6 months mortality compared to PCT. Wen *et al*. [40] found significantly higher levels of presepsin in nonsurvivors compared to survivors and no significant differences in PCT levels between the two outcome groups, which is in line with our results. They reported presepsin levels, lactate levels, and SOFA score as risk factors for patients’ in-hospital mortality from sepsis. Also, they did not find an association between PCT levels and APACHE II score with in-hospital mortality prediction.

This study has several limitations. First, the sample size is relatively small, although several studies have a similar or smaller sample size. Second, the method of measurement of presepsin used in our study limited the comparison of cutoff values of presepsin with one of other studies in which presepsin values were measured using a compact, automated immunoanalyzer, PATHFAST, based on a chemiluminescent enzyme immunoassay. Third, predictive value of PCT for mortality from sepsis was not analyzed, so we could not compare mortality prediction ability of presepsin with the one of PCT.

The present study has some strength as well. First, it was a study conducted in two university centers. Second, patients included in the study were suspected and diagnosed by a specialist in infectious diseases. Third, levels of presepsin and other tested biomarkers were monitored four times during study.

## Conclusions

Presepsin has a good ability to predict mortality. Initial presepsin concentrations better reflect poor disease outcome compared to presepsin concentrations 24 and 72 hours after admission. The lack of association between PCT values and disease outcomes may question the prognostic value of PCT.
